# Attention induced neural response trade-off in retinotopic cortex under load

**DOI:** 10.1038/srep33041

**Published:** 2016-09-14

**Authors:** Ana Torralbo, Todd A. Kelley, Geraint Rees, Nilli Lavie

**Affiliations:** 1 Institute of Cognitive Neuroscience, University College London, London, UK; 2 Wellcome Trust Centre for Neuroimaging, University College London, London, WC1N 3BG, UK

## Abstract

The effects of perceptual load on visual cortex response to distractors are well established and various phenomena of ‘inattentional blindness’ associated with elimination of visual cortex response to unattended distractors, have been documented in tasks of high load. Here we tested an account for these effects in terms of a load-induced trade-off between target and distractor processing in retinotopic visual cortex. Participants were scanned using fMRI while performing a visual-search task and ignoring distractor checkerboards in the periphery. Retinotopic responses to target and distractors were assessed as a function of search load (comparing search set-sizes two, three and five). We found that increased load not only increased activity in frontoparietal network, but also had opposite effects on retinotopic responses to target and distractors. Target-related signals in areas V2–V3 linearly increased, while distractor response linearly decreased, with increased load. Critically, the slopes were equivalent for both load functions, thus demonstrating resource trade-off. Load effects were also found in displays with the same item number in the distractor hemisphere across different set sizes, thus ruling out local intrahemispheric interactions as the cause. Our findings provide new evidence for load theory proposals of attention resource sharing between target and distractor leading to inattentional blindness.

Much research has established that people’s ability to focus attention effectively on their task, in the presence of irrelevant distractors, depends not only on the extent to which the distractors are presented away from the current focus, but also on the level of perceptual load in the task. Irrelevant distractors appearing in the periphery, clearly separate from the attended task, consistently elicit visual cortex responses in tasks of low perceptual load (e.g. detection of a ‘target’ item, that can pop out from among dissimilar items). In contrast tasks of high perceptual load (e.g. requiring complex discrimination of a target based on specific shape-colour combinations) are associated with significantly reduced visual cortex response to any irrelevant distractors (e.g. refs 1, 2, 3; see ref. 4 for a recent review) resulting in the experience of ‘inattentional blindness’. These effects are found across different manipulations of load, throughout visual cortex, from occipital cortex including primary visual cortex area V1, the superior colliculus and LGN (e.g. refs 1,3,5 and 6), to cortical areas involved in complex image recognition (e.g. refs 2 and 7). Additional studies have demonstrated the behavioural significance of these findings, showing that distractors produced significant interference in task performance in conditions of low but not high perceptual load (e.g. refs 8, 9, 10, 11, 12) whereby people report experiencing ‘blindness’ to unattended stimuli instead^13^,^14^,^15^. All these findings are accounted for by the load theory of attention and cognitive control (e.g. refs 10 and 16). In load theory neural resources mediating perception are of limited capacity, and therefore depend on the allocation of attention in tasks of high perceptual load which consume full capacity. In addition, the theory proposes that although perceptual processing resources are of limited capacity they are spent to the full, thus resulting in perception of all stimuli within capacity whether task-relevant or irrelevant. Therefore tasks of low perceptual load result additionally in the perception of irrelevant distractors.

While research into the effects of perceptual load on unattended distractor processing provides strong support for Load Theory, the neural mechanisms mediating these effects require further elucidation. Specifically, the attribution of load effects on distractor processing to the level of demand on capacity placed by the attended task leads to the prediction that level of neural response to the distractor should be inversely related to the level of neural activity associated with the attended task processing. In other words, brain activity measured during performance of a selective attention task is expected to show a load related neural response trade-off between the attended and unattended processing, reflecting the sharing of limited resources. The hypothesis of a resource sharing resulting in a response trade-off further leads to the prediction that the magnitude of load effect on unattended distractor signal should be proportional to the magnitude of load effect on the attended target signal. Thus a gradual increase in the level of load in an attended task should be associated with a gradual increase in the target signal, but importantly a concomitant gradual decrease in the distractor signal of a similar magnitude to increase in the target signal with load.

Moreover, a core claim of perceptual load theory is that perceptual load leads to ‘early selection’ reducing elementary perceptual processing associated with contrast detection for irrelevant distractors (e.g. ref. 14) and the related neural signals in retinotopic primary visual cortex areas (both striate and extrastriate, e.g. refs 3,17 and 18) and thus leading to inattentional blindness^13^,^14^,^15^. This suggests that a load-induced trade-off in neural activity should be found already in the retinotopic visual cortex response to targets versus distractors. This trade-off in neural responses would explain why people fail to detect unattended stimuli and therefore report inattentional blindness under high perceptual load.

To test this hypothesis, we varied the level of load in a visual search task in a gradual manner, by changing the search set size and comparing search tasks involving two, three or five search items. Participants performed the search task under these different levels of perceptual load while ignoring two irrelevant distractor checkerboards in the periphery (see Fig. 1). Retinotopic and functional localizer mapping allowed us to measure the distinct retinotopic responses related to the target (search array) and to the distractor (checkerboard) stimuli in areas V1–V3, and we assessed these as a function of the gradual increase in search load with increased set size.

## Results

### Behavioural results

Alpha levels were set at p < 0.05 for significance. Participants’ mean response time (RT) and accuracy rates were submitted to repeated measures ANOVAs Greenhouse-Geisser corrected for sphericity (degrees of freedom are rounded to the nearest integer value). Repeated measures ANOVAs of the visual search task response times (RT) as a function of set size (two, three or five) revealed that increased search set size was associated with longer response times (F(1, 20) = 96.74, MSE = 3491, p < 0.001, ηp2 = 0.858, see Fig. 2), as expected. The effects were significant for both the increase from set size two to three (t(16) = 8.32, p < 0.001, Cohen’s d = 2.02; two tailed t-test as in all other results reported, unless specified otherwise) and from set size three to five (t(16) = 8.96, p < 0.001; Cohen’s d = 2.17). Figure 2 illustrates a linear effect of set size. To formally examine this, we ran a linear mixed model analysis on the individual trial response times with the factors of set size and set size^2^ as the linear and quadratic predictors respectively, and participant as random effect (to account for the individual participant RT slope). This analysis established a significant linear trend for set size, and no effect of the nonlinear predictor (set size predictor: estimate = 115.75 (SE = 27.87), t = 4.153, p < 0.001; quadratic predictor: estimate = −5.83, (SE = 3.80), t = 1.53, p = 0.12). Model comparison revealed no significant differences between linear models that include or did not include a quadratic predictor, χ^2^ (1) = 2.35, p = 0.12. These results confirm that increased set size led to a linear increase in the demand on search capacity, as expected for this conjunction search task (e.g. ref. 19).

A similar ANOVA on the search errors replicated the effect seen on RT, demonstrating a significant increase in search errors with increased set size (F(1, 20) = 59.06, MSE = 20.23, p < 0.001, ηp2 = 0.78). As in the RT results, this effect was significant for both the increase in set size from set size two to three (t(16) = 4.34, p = 0.001, Cohen’s d = 1.05) and the set size increase from three to five (t(16) = 8.20, p < 0.001, Cohen’s d = 1.99).

Eye-monitoring data was analysed for fourteen of the participants (three were excluded due to contact lenses artefacts) (Fig. S1). Repeated measures ANOVAs on the average eye position with the factor of set size showed no effect of set size on either horizontal (F(2, 26) = 1.31, p = 0.286, or vertical distance from fixation F(2, 26) = 1.15, p = 0.330. There was also no effect on the standard deviation of the distances (F(2, 26) = 1.26, p = 0.29 for horizontal; F < 1 for vertical distance). Thus none of the effects of set size can be attributed to differences in eye fixation.

### Whole brain analysis

**Figure 1:**
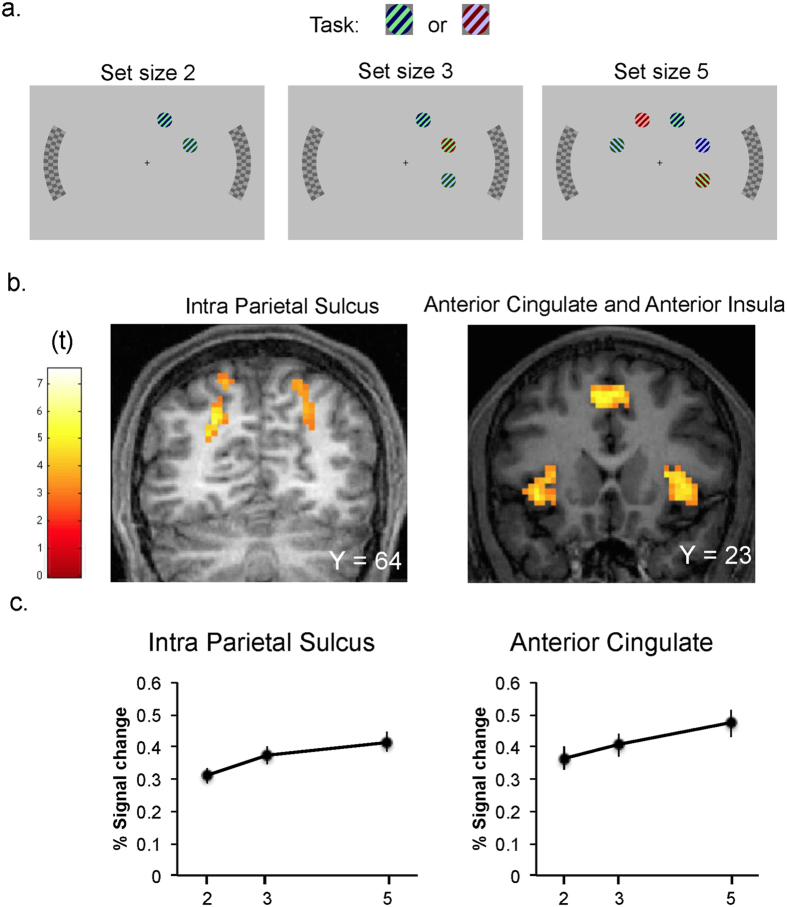
Example task displays in each conditions of set size (**A**) and full-brain activity results (**B,C**). As shown in panel A set size two always included two items within the same quadrant (equally likely in any of the four possible quadrants). Set size was increased by adding search items in adjacent positions to those in the lower set size displays (within the same hemifield for set size three) while set size five displays had either two or three items in each hemifield (see also Fig. 4). There was only one target in every trial that appeared equally likely in any of the eight possible locations in all set sizes. Panel B shows the regions found to have increased activity as a function of set size and Panel C shows the set size effect on percentage signal change in each of these regions.

**Figure 2:**
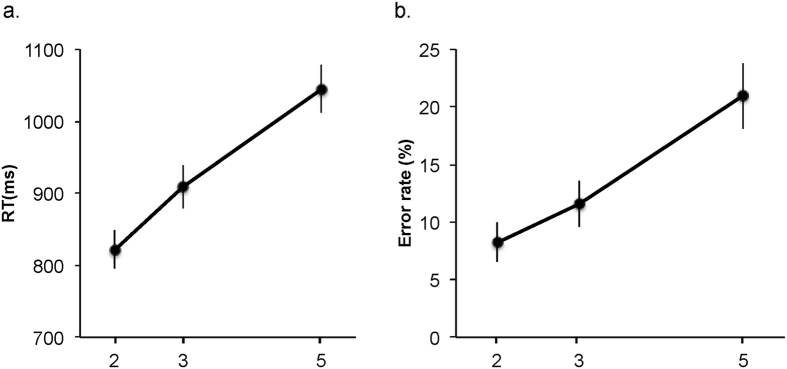
Behavioural performance. Participants mean response time (panel A) and error rates (panel B) as a function of load. Bars depict SEM.

Increased set size was associated with significant increases in activity in the frontoparietal attention network, including bilateral intraparietal sulcus, bilateral insula, left middle frontal gyrus, anterior cingulate cortex (p < 0.005 uncorrected voxel level, p_FWE-corr_ < 0.05 at cluster level, Fig. 1). These regions are consistent with regions typically associated with the effects of attentional load in a variety of tasks (e.g. refs 3,7,20, 21, 22). Additionally, a polynomial regression, testing for both linear and quadratic trends over the full brain indicated a significant linear effect for the effect of load on the frontoparietal network (p < 0.005 uncorrected voxel level, p_FWE-corr_ < 0.05 at cluster level; Table 1), consistent with the linear effects of load found on RTs. No regions showed a significant quadratic trend once the variance associated with a linear component was taken into account (all p_FWE-corr_ > 0.12). These results concur with previous findings that gradual increases in load through increased set size are associated with a linear increase in frontoparietal^21^,^23^,^24^,^25^ in the range of set size increase up to five items as reported here.

#### Retinotopic responses to target and distractor ROI

**Figure 3:**
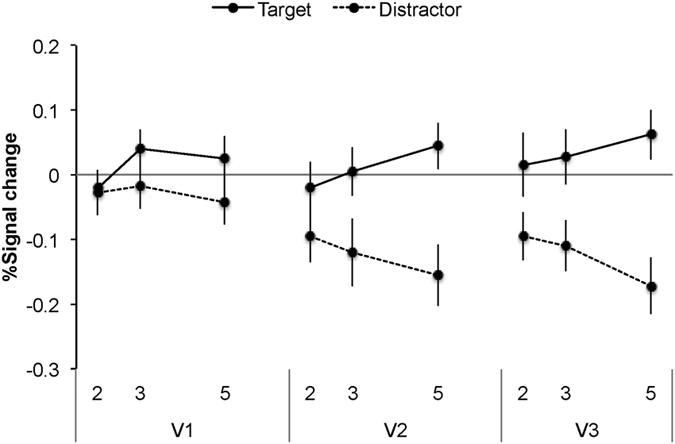
Neural response in target and distractor retinotopic regions as a function of search set size for V1, V2 and V3.

Our main hypothesis concerned the effects of load on retinotopic responses to the target versus distractor items. Participants beta values corresponding to retinotopic locations of target or distractor items were submitted to repeated measure ANOVAs with attention (target, distractor) and search set size (two, three, five) as factors for V1, V2, and V3/VP (Fig. 3; see also Supplementary Information). The ANOVAs revealed a significant main effect of attention in all three areas F(1, 16) = 6.11, MSE = 0.518, p = 0.025, for V1; F(1, 16) = 20.32, MSE = 1.17, p < 0.001, for V2; F(1, 16) = 16.71, MSE = 2.45, p = 0.001 for V3). As shown in Fig. 3, BOLD signals were higher in the target ROIs than in the distractor ROIs for all areas despite the fact that the distractor stimulus was substantially larger than the target stimulus, even when cortical magnification was taken into account. Importantly there was an interaction between attention and set size in V2: F(2, 32) = 15.28, MSE = 0.164, p < 0.001, and V3: F(2, 32) = 12.53, MSE = 0.248, p < 0.001. The interaction with set size was not significant in V1 (F(2, 32) = 2.22, MSE = 0.16, p = 0.12), however the effect of perceptual load on V1 activity was found when target and distractor signals were compared between the low load (set size two) and high load (set size five) level (t(16) = 2.01, p = 0.030, one tailed). As can be seen in Fig. 3, load affected both the distractor and the target signals in a resource trade-off manner in areas V2–V3: while distractor-related signals were linearly reduced with increased load, target-related activity linearly increased with higher load. This was confirmed in a polynomial regression on the distractor and target ROIs showing a significant linear effect of set size both on the distractor responses (average parameter estimate for the linear effect = −0.164, SEM = 0.054, t(16) = 3.08, p = 0.003 one tailed for V2; average parameter estimate = −0.216, SEM = 0.069, t(16) = 3.19, p = 0.002 one tailed for V3) and on the target responses (average parameter estimate for the linear effect = 0.143, SEM = 0.062, t(16) = 2.36, p = 0.01, one tailed for V2) with a similar trend that approached significance in V3 (average parameter estimate for the linear effect = 0.151, SEM = 0.09, t(16) = 1.57, p = 0.06, one tailed). Moreover, as also shown in Fig. 3 the increase in target response with increased set size was of a similar magnitude to the reduction in distractor response with increased set size (the average slope for increase in target signal with increased set size was 0.20 for V2 (SEM = 0.09) and 0.18 for V3 (SEM = 0.08) while the average slope for decrease in distractor signal with set size load was 0.19 for V2 (SEM = 0.07) and 0.23 for V3, (SEM = 0.08), t < 1 for slope comparisons in both areas).

Overall, these findings demonstrate a resource trade-off between retinotopic activity related to attended targets compared to unattended distractors. This resource trade-off supports our load theory prediction that reduced distractor processing is inversely related to the draw on capacity for target processing. The findings that perceptual load induces a resource trade-off in the response to attended versus unattended stimuli already in retinotopic cortex is as we predicted on the basis of load theory claim that perceptual load effects lead to ‘early selection’.

### Connectivity

To examine whether perceptual load modulated the functional connectivity between the frontoparietal regions associated with a main effect of load and the target and distractor ROIs in V2 and V3 we have conducted a psychophysiological interaction analysis (see Supplementary Material). This analysis revealed that the manipulation of set size was associated with increased functional connectivity between the distractor V3 ROI (representing the left and right checkerboards) and the left inferior frontal gyrus (Z = 4.37, p_FWE_ = 0.001; MNI coordinates −33, 2, −14; Number of voxels = 109). These findings further support the load hypothesis in showing that increased perceptual load is associated with greater connectivity between attentional control regions typically associated with the main effect of load and the level of distractor signal in retinotopic cortex. Furthermore, the specific pattern reflected greater connectivity between a lower distractor signal (average beta values for the composite distractor V3 ROI were −0.71, −0.84, −1.27 for set sizes two, three and five respectively) and inferior frontal cortex as set size was increased. This pattern suggests an attention suppression effect on retinotopic distractor responses with increased set size that is potentially mediated by increased functional connectivity with frontal attentional load associated areas, in support of our load hypothesis.

### Local interactions

**Figure 4:**
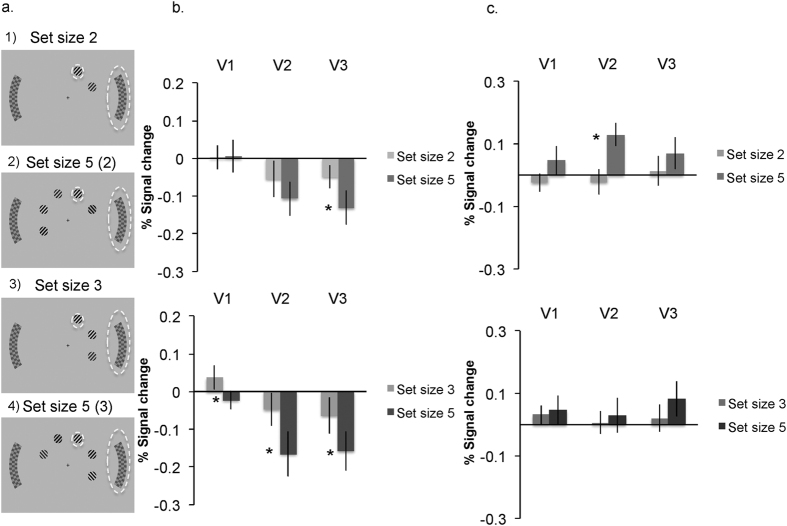
Examples of experiment conditions and response in distractor retinotopic regions as a function of set size for displays with the same number of items in the distractor hemifield. Panel (A) shows the distractor checkerboard signal that was entered into analysis (marked with a dashed ellipse, while the target is marked with a dashed circle (for figure illustration only). Set size five trials were split into five (two) or five (three) according to the number of search items in the distractor hemifield. Search gratings were coloured (as in Fig. 1). Panel (B) shows the effects of set size on distractor response in V1–V3, for trials with the same number of items within the distractor hemifield (where the increased set size involves more items in the opposite hemifield only). Asterisks denote statistical significance at p < 0.05. Results are shown for the left and right checkerboards combined. Panel (C) shows the effects of set size on the target response in V1–V3.

To ensure that the set size effects on the unattended distractor processing are due to a draw on attention capacity as we claim, rather than any form of local interactions, we reanalysed the set size effect on the distractor signals, for trials with the very same set size in the distractor hemifield across the different set sizes. The distractor ROI signals entered into this analysis were those from displays in which the increased set size only affected the opposite hemifield to the distractor ROI (see Fig. 4). For this analysis the signal in set size two condition was compared with the signal from a sub-set of the data in the set size five displays that had two items in the distractor hemifield (on the right in this example) and three items in the opposite hemifield (on the left); while a comparison of set size three with set size five involved the set size five displays that had three items in the distractor hemifield (on the right) and two items in the opposite hemifield (on the left, see Fig. 4). In this way the effect of set size concerned the very same number of items within the distractor hemifield (Fig. 4, Panel b). The results replicated the same pattern found overall: distractor responses were reduced in set size five compared to set size three in all areas (t(16) = 2.77, p = 0.014 in V1; t(16) = 4.16, p < 0.001 in V2; t(16) = 2.40, p = 0.028 in V3) and compared to set size two in V3 (t(16) = 2.77, p = 0.014; t < 1 in V1 and t(16) = 1.82, p = 0.086 in V2, although the lack of significance is likely due to the reduced power with smaller number of hemifield set size matched trials). In addition, Fig. 4 (panel c) shows that the increase in the target signal with set size is also found for the same number of items in the target hemifield across set sizes (so that the increased number of items with set size just concerns the opposite hemifield), though these effects reached significance only for set size 5 compared to set size 2 in V2 t(16) = 3.04, p = 0.008 (t(16) = 1.33, p = 0.2 for V1; t(16) = 1.26, p = 0.2 for V3, t < 1 for the comparisons of set size five and three). Finally the set size five condition also allowed us to compare the distractor signal as a function of the increase in the number of items within the same or within the opposite distractor hemifield only, for the same level of overall load (set size 5 displays: Fig. 4 panel A, comparison of signal in displays 2 versus 4 for distractor in the same or opposite hemifield as the target). This analysis showed no significant differences in distractor suppression as a function of the number of items within the same distractor hemifield (t(16) = 1.573, p = 0.135 in V2; t < 1 for V1, V3) or in the opposite hemifield to the distractor (t(16) = 1.37, p = 0.188 for V2, t < 1 for V1, V3).

These findings confirm our hypothesis that the reduced distractor response is due to load on attention, rather than local (hemisphere-specific) suppression. These results also show that load-dependent distractor modulations must originate in higher regions, where representations are not confined to a hemifield, in line with the whole brain findings of frontoparietal activity related to load and with the functional connectivity results.

**Figure 5:**
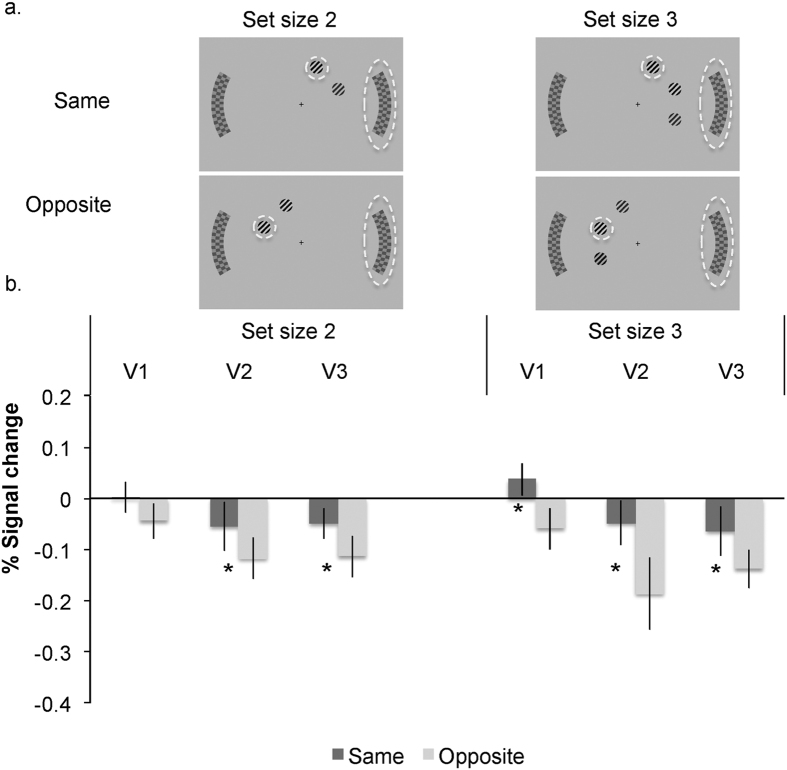
Display examples and response in distractor retinotopic locations shown per set sizes two-three and distractor and target within the same or opposite hemifields. Panel (A) shows the distractor signal entered into analysis (marked with a dashed ellipse, while the target is marked with a dashed circle (for figure illustration only). Panel (B) shows the effect of set size (two and three) on distractor response in V1–V3 for displays with distractor and search items either within the same or in opposite visual fields. Asterisks denote significance at p < 0.05. Results for the left and right checkerboards are combined.

Local interactions were examined further by comparing the distractor signal between trials in which the search items were all presented within the distractor hemifield and those in which all the search items were all in the opposite hemifield. This analysis could only be run for set sizes 2 and 3 (since set size 5 always included a mixture of items in each hemifield). The results indicated a higher distractor signal when the search items were within the same hemifield as the distractor compared to between hemifield (For set size 3: t(16) = 2.70, p = 0.015 in V1; t(16) = 3.79, p = 0.002 in V2; t(16) = 2.42, p = 0.028 in V3. For set size 2: t(16) = 1.92, p = 0.07 in V1; t(16) = 3.62, p = 0.002 in V2; t(16) = 4.012, p < 0.001 in V3; Fig. 5).

Furthermore an ANOVA that included both factors of hemifield and set size (for the data shown in Fig. 5) revealed no interaction of the effect of increased set size and hemifield (F(1, 16) = 2.32, p = 0.147 for the interaction in V1, F(1, 16) = 2.74, p = 0.117 for the interaction in V2, F < 1 for the interaction in V3). These findings clearly rule out local suppressions of the distractor or target signal as the source for the effect of set size. Instead they support the attention spill over hypothesis (e.g. ref. 8) in which attention spills over to distractor stimuli in lower set sizes (e.g. refs 9 and 26) leads to distractor processing. Such spill over favours distractor items near the target compared to those further away^27^.

#### General discussion

The present study established the effects of gradual increases in perceptual load both on the frontoparietal attention network, consistent with previous research (e.g. refs 3,7,20, 21, 22). More importantly, we extended these findings to characterise the distinct retinotopic response to attended targets versus unattended distractors. Our findings indicate a resource sharing pattern in retinotopic cortex V2–V3: gradual increases in perceptual load through increased search set size were associated with both increased retinotopic V2–V3 responses related to the attended target stimuli and reduced response associated with the unattended distractor stimulus. Moreover, the specific findings that reduction in distractor related activity was of the same magnitude as the increase in attended target signal with increased load clearly lends itself to a neural resource trade-off interpretation, as we predicted.

Overall, these results provide a novel line of support for the core claims of load theory that reduced distractor processing is the result of increased demand on perceptual capacity in the attended task and that the effects of perceptual load lead to ‘early selection’, being found already at the retinotopic visual cortex response to attended targets versus unattended distractors. The effects on retinotopic responses to distractors (as compared with targets) were found already in V1 as well as V2–V3 and these effects can also account for previous behavioural findings that increased perceptual load leads to inattentional blindness: failing to notice a distractor in conditions of higher perceptual load in the task (e.g. refs 15 and 28). The finding that inattentional blindness under load is due to reduced detection sensitivity, with no effects on decision criterion in high perceptual load (e.g. refs 13 and 14) can be explained by a reduced signal to distractor in retinotopic visual representations in striate and extrastriate cortex.

The specific pattern of a load induced trade-off in the neural signal established here concurs with a few previous demonstrations of ‘push-pull’ effects of attention on visual cortex responses to target and distractors, and suggests that a neural resource trade-off is mediating these effects. For example, Somers *et al.*^29^ found spatially specific enhancement and spatially specific suppression related to the attended versus unattended locations respectively in retinotopic areas V1–V3. Moreover, paying attention (in order to report memory post-exposure) to images of either faces or scenes leads to both enhancement and suppression of the related face or scene signal in category selective areas of visual cortex^30^. However these earlier studies did not vary demands on target processing (rather they varied which location or which image category was attended) and thus the general ‘push pull’ pattern can only be suggestive of capacity sharing in perception related processing. Pinks *et al.*^7^ studied more directly the effects of attention in relation to the level of demand on perceptual capacity. They compared the level of activity related to target and distractor pictures of meaningful objects (such as fruits) as a function of the perceptual demands in the attended task. Their results indicated a push-pull effect of attention on the V4/TEO response to targets and distractors. In addition, their effect was found across the two hemispheres (e.g. increased signal in right V4 for attended targets in the left visual field together with reduced signal in left V4 to unattended distractors in the right visual fields) consistent with the cross-hemispheric effects we report (in the Local Interaction results section) in addition to the within-hemisphere effects we also establish. These effects were not apparent in V1–V3/VP in Pinsk *et al.*’s study however this may be due to reduced task sensitivity to reveal these (e.g. the use of meaningful images which required object recognition may have been particularly suited to attentional modulations of V4/TEO where object recognition is typically implicated). Furthermore since all the previous studies that compared attended and unattended activity did not gradually increase the level of load in the attended task, a resource trade-off function could not be previously plotted.

Thus, the present results significantly extend previous research to now demonstrate a specific pattern of push-pull effects of attentional load in areas V2–V3 both within and across hemispheres. Moreover, our demonstration that the search set size slopes related to gradual increases in task load were of corresponding magnitude for both the increase in target signal and the decrease in distractor signal can more tightly support the conclusion that the effects of attention load are due to a resource trade-off in neural responses. Additionally, the findings that distractor ROI shows greater functional connectivity with the inferior frontal gyrus with higher load suggests a load-mediated attention control over retinotopic cortex responses to distractor in further support of our hypothesis.

As expected from the load hypothesis and the load mediated frontal-retinotopic connectivity related to the distractor response, our local interaction analyses further demonstrated that the reduced distractor response with increased set size is associated with load on attention, rather than local suppression (e.g. due to crowding, or greater suppressive interactions within receptive fields, c.f. ref. 31) of distractor signals within the same hemifield as the target. In addition, our finding that in conditions of low load distractor signal was greater when the search items were in the same hemifield rather than in the opposite hemifield not only rules out local suppression effects but also provides novel evidence for load theory claim of a ‘spill over’ of resources in conditions of low load in the attended task (see also ref. 32 for supportive findings), which favours items in the vicinity of the target^27^.

These findings are consistent with a large volume of previous demonstrations of perceptual load effects that cannot be accounted for by local suppressions, or hemisphere specific interactions (for instance because the effect of load is varied at fixation, without any increase in the number of items, e.g. refs 1,3 and 33).

The apparent inconsistency with a recent finding that increased perceptual load only affected behavioural response-competition effects from flankers in the opposite hemifield to the target^34^ can be explained by the null flanker effects found for flankers within the target hemifield already in the low load conditions. Clearly load cannot cause any reduction in distractor effects when these are already at floor in the low load conditions.

Of course the findings do not rule out other effects of hemispheric interactions e.g. perhaps due to hemisphere-specific resources in the case of motion tracking^35^. Instead they reinforce load theory claims that distractor processing depends on the level of draw on perceptual capacity (across different manipulations, which are typically not hemisphere specific).

Finally, it is worth noting that the linear increases in fronto parietal BOLD signal related to attentional load may be restricted to our use of a set size of up to five items within our conjuction search task. Larger set size could perhaps lead to an asymptote in the effect on the neural signal, indicating resource saturation. For example Mitchell and Cusack^25^ report a saturation of the set size effect with a set size of eight items. Such saturation once found for a target processing is also predicted to lead to an asymptote in the distractor response signal (showing no further decline with the higher set sizes where the asymptote is found). Testing these predictions should prove an interesting avenue for future research.

## Materials and Methods

### Participants

The research was approved by the UCL ethics committee and was carried out in accordance with the approved guidelines. Eighteen participants (12 females; age: 19–31, mean 25 years old; all right-handed) were recruited in keeping with the standard sample size in attention research. The data from one participant (female) could not be analysed, due to excessive head motion in the scanner. None of the participants reported past history of psychiatric or neurological diseases, and all gave informed written consent. Participants had normal or corrected-to-normal visual acuity.

### Stimuli and Task

Stimuli were presented on a uniform middle grey background. A small cross (0.38° × 0.38°) appeared in the centre of each display to mark fixation. Search stimuli were oriented gratings subtending 1.1° that appeared at 3.7° from the fixation cross, and could be tilted 45° or 315°. Orientation was randomly chosen for each grating on every trial. The distance between patches was 2.8° (center to center). Two black and white checkerboards (1.5° width × 7° height) appeared on the left and right sides of each display with their centre positioned 7.4° away from fixation. The colour set for the search stimuli was: light green (0.51, 0.9, 0.51), green (0, 0.314, 0), blue (0, 0, 0.392), maroon (0.471, 0, 0), lilac (0.725, 0.725, 1) and pink (1, 0.588, 0.588). The search targets were defined as having either the combination of light green and blue or lilac and maroon (Fig. 1). Search “non-target” gratings had the combinations of light green and dark green, light green and maroon, lilac and dark green, lilac and blue, pink and dark green, pink and blue, pink and maroon.

Participants’ task was to discriminate which of the two target gratings appeared while ignoring the checkerboard distractors. The search task had either two, three or five gratings always appearing in adjacent locations (Fig. 1). As shown in Fig. 1 set size two always included two items within the same quadrant (equally likely in any of the four possible quadrants). Set size was increased by adding search items in adjacent positions to those in the lower set size displays (within the same hemifield for set size three) while set size five displays had either two or three items in each hemifield (see also Fig. 4). There was only one target in every trial that appeared equally likely in any of the eight possible locations in all set sizes. Participants were instructed to respond to the presence of any of the targets as accurately and quickly as possible while keeping their eyes fixated at the centre marked with a small cross on each display. Each search trial started with a fixation cross in the centre of the screen during 500 ms followed by a search display. Search stimuli remained on the screen during 200 ms, and the peripheral checkerboards flickered after 100 ms. In null trials only the fixation cross was present. A blank interval of variable duration followed so the intertrial interval was 3, 5, or 7 seconds. Response was collected during 2 seconds.

Participants completed 4 scan sessions each composed of 87 trials (24 trials per each of the search set size plus 15 null trials). Search set size, target location and inter-trial interval were fully counterbalanced within each of the four scanning sessions. The two target gratings were fully counterbalanced with set size, target location and ITI value across the whole experiment. All trials were presented in random order. Each scan lasted 480 seconds and started with a 10 volumes blank period. The initial 5 volumes were considered as dummy scans and not included in any analysis. Stimuli were viewed via a mirror system mounted on the head coil. The experiment was programed in MATLAB with Cogent toolbox used for graphical presentation (http://www.vislab.ucl.ac.uk/cogent.php). Mixed model analysis on the individual behavioural responses was estimated in R^36^ using the lme4^37^ and lmerTest^38^.

Following the scanning experiment, participants underwent two runs of functional localizers blocks and two runs of retinotopic mapping scans (see Supplementary information). To ensure whether participants maintained fixation eye movements were controlled during the main experiment with a long-range optics ASL 5000 Eye Tracking System. Eye position was continuously sampled at 60 Hz in every trial. Calibration was performed immediately before the experiment once the participants’ head was still and in a good position for scanning. The luminance conditions during calibration were the same as the experiment.

#### MRI acquisition and analysis

Scanning was performed at the Wellcome Trust Centre for Neuroimaging at UCL. All images were acquired using a Siemens 3T Allegra MRI scanner with a single channel head coil. Anatomical images were acquired using a T1-weighted sequence with 1mm voxel resolution images (176 sagittal slices, echo time = 2.4 msec; acquisition time per slice = 7.92 msec, matrix size = 256 × 240, flip angle = 15°). Whole-brain echoplanar functional images were acquired in 38 transverse slices (repetition time = 2.28 s, matrix size = 64 × 72, slice thickness = 2 mm with 1 mm gap, voxel size 3 mm, descending slice acquisition). In the main experiment, 4 scans of 210 volumes each were acquired. Then two additional scans of 110 volumes were acquired to be used as functional localizers as well as two more scans of 165 volumes for retinotopic mapping. All scans included 5 dummy volumes at the start to allow for T1 equilibration effects. A field map was acquired to assist in unwarping the functional images.

All data were processed with SPM8 (http://www.fil.ion.ucl.ac.uk). Functional images were slice time corrected, realigned with a representative functional volume and unwarped. Additionally the main experiment images were morphed into the standard Montreal Neurological Institute space via the unified-segmentation procedure (only for whole-brain analysis; in two participants a new AC origin was set to improve alignment) and spatially smoothed using a 4 mm full-width at half maximum Gaussian kernel. Retinotopy and localizer images were preserved in their native space (and not smoothed). A high-pass filter (cut-off, 128 sec) was applied to the data for removing low-frequency signal drifts. All events representing the presence of a target in any of the eight possible search locations under the three different set sizes besides null trials were modelled using a general linear model. Each event was modelled as an impulse of activity at the onset of the search display and was convolved with the canonical hemodynamic response function in SPM8. Statistical parametric maps were obtained using t contrasts testing for a greater activity in set size 5 compared to set size 2 for each participant. In a second stage, statistical parametric maps were generated at group level where contrast images for each participant in the condition of interest were submitted to one sample t-test. SPM maps were generated using a voxel-level threshold of p < 0.005 (uncorrected) and a family-wise error correction at cluster level of p < 0.05. Additionally, load was modelled as a continuous variable in a polynomial regression where trials were weighted by their set size. Statistical parametric maps were obtained for each participant for the activations that followed either a linear of quadratic trend. In a second stage, statistical maps were generated at group level using a t-contrast on the contrast images obtained from each subject.

Anatomical images were co-registered with a mean functional image and used for surface reconstruction, which was performed with the Freesurfer image analysis software (http://surfer.nmr.mgh.harvard.edu/). Data from the retinotopic mapping was used to define the borders between areas V1, V2 and V3/VP in each hemisphere^39^. Data from the localizer scans were used to define the ROIs within each visual area that responded more strongly to each stimulus location (see Supplementary Material). Functional data from both scans were projected onto flattened representations of each participant’s visual cortex. ROIs corresponding to the search grating locations or checkerboard distractor locations in each visual area were drawn in Freesurfer and the ROIs created were then imported into SPM8.

Beta weights (parameter estimates) corresponding to the events of interest (i.e. a target present in each search location, a distractor checkerboard present on the left or right) were obtained for every ROI for each participant, using Marsbar (http://marsbar.sourceforge.net/) and then averaged across the ROIs in each visual area for each experimental condition. Distractor related signal was extracted separately for the ventral and dorsal sections in each of the V1–V3 ROIs and than averaged across sections and across left and right ROIs. In the local interactions analysis, events were modelled for target presence in either the right or left hemifield, for each set size (Fig. 4, Panel b) and in another regression for targets presence in each of the target locations for each set size (Fig. 4, Panel c). Beta weights were obtained for every distractor ROI separately for trials with target in the same or opposite hemifield as the distractor for each set size (Fig. 4, Panel b). Beta weights were also obtained for every target ROI separately for trials with different number of items in the target hemifield for each set size (Fig. 4, Panel c). Percent signal change was computed in Marsbar with the function event_signal for illustration purposes only. All statistical analysis were performed using the beta values (parameter estimates) obtained from the GLM.

## Additional Information

**How to cite this article**: Torralbo, A. *et al.* Attention induced neural response trade-off in retinotopic cortex under load. *Sci. Rep.* **6**, 33041; doi: 10.1038/srep33041 (2016).

## Supplementary Material

Supplementary Information

## Figures and Tables

**Table 1:** MNI coordinates of significant clusters in each whole brain contrast; p < 0.005 voxelwise uncorrected and p_FWE-corr_ < 0.05 cluster-wise corrected for multiple comparisons.

Contrast	Hemisphere	Region	MNI Coordinates (x, y, z)	Z score	Number of voxels in cluster
Increased activity with load (−1 0 1)	Right	Anterior Insula	33, 26, −2	4.86	155
	Left	Intraparietal Sulcus	−21, −67, 40	4.48	102
	Left	Middle Frontal gyrus	−24, −4, 52	4.22	51
	Left	Anterior Insula	−30, 26, 1	4.15	97
	Middle	Anterior Cingulate Cortex	6, 14, 49	4.12	93
	Right	Intraparietal Sulcus	24, −61, 40	3.51	60
Linear trend in polynomial regression	Right	Anterior Insula	33, 26, −2	5.16	156
	Left	Intraparietal Sulcus	−21, −67, 40	4.62	99
	Left	Anterior Insula	−39, 17, −5	4.16	92
	Left	Middle Frontal gyrus	−21, −4, 55	4.14	54
	Middle	Anterior Cingulate Cortex	−9, 14, 46	3.95	99
	Right	Intraparietal Sulcus	21, −49, 43	3.70	71

Coordinates refer to the location of maximal activation.

## References

[b1] Rees G., Frith C. & Lavie N. Modulating irrelevant motion perception by varying attentional load in an unrelated task. Science 278(5343), 1616–1619 (1997). 9374459 10.1126/science.278.5343.1616

[b2] Yi D. J., Woodman G. F., Widders D., Marois R. & Chun M. M. The neural fate of ignored stimuli: Dissociable effects of perceptual and working memory load. Nat Neurosci 7(9), 992–996 (2004). 15286791 10.1038/nn1294

[b3] Schwartz S. *et al.* Attentional load and sensory competition in human vision: modulation of fMRI responses by load at fixation during task-irrelevant stimulation in the peripheral visual field. Cereb. Cortex 15, 770–786 (2005). 15459076 10.1093/cercor/bhh178

[b4] Lavie N., Beck D. M. & Konstantinou N. Blinded by the load: attention, awareness and the role of perceptual load. Philos T Roy Soc B 369, 20130205 (2014). 10.1098/rstb.2013.0205 PMC3965161 24639578

[b5] Bahrami B., Lavie N. & Rees G. Attentional load modulates responses of human primary visual cortex to invisible stimuli. Curr. Biol. 17(6), 509–513 (2007). 17346967 10.1016/j.cub.2007.01.070 PMC1885953

[b6] O’Connor D. H., Fukui M. M., Pinsk M. A. & Kastner S. Attention modulates responses in the human lateral geniculate nucleus. Nat Neuroscience 5, 1203–1209 (2002). 12379861 10.1038/nn957

[b7] Pinsk M. A., Doniger G. M. & Kastner S. Evidence for a push-pull mechanism of selective attention in human extrastriate cortex. J. Neurophysiol 92, 622–629 (2004). 14973320 10.1152/jn.00974.2003

[b8] Lavie N. Perceptual load as a necessary condition for selective attention. J. Exp. Psychol. Hum. Percept. Perform 21, 451–468 (1995). 7790827 10.1037//0096-1523.21.3.451

[b9] Lavie N. & Cox S. On the efficiency of attentional selection: Efficient visual search results in inefficient rejection of distraction. Psychol Sci 8, 395–398 (1997).

[b10] Lavie N., Hirst A., De Fockert J. W. & Viding E. Load theory of selective attention and cognitive control. J. Exp. Psychol: Gen 133, 339–354 (2004). 15355143 10.1037/0096-3445.133.3.339

[b11] Forster S. & Lavie N. High perceptual load makes everybody equal: Eliminating individual differences in distractibility with load. Psychol Sci 18(5), 377–382 (2007). 17576274 10.1111/j.1467-9280.2007.01908.x

[b12] Forster S. & Lavie N. Failures to ignore entirely irrelevant distractors: the role of load. J. Exp. Psychol. Appl. 14, 73–83 (2008). 18377168 10.1037/1076-898X.14.1.73 PMC2672049

[b13] Carmel D., Thorne J., Rees G. & Lavie N. Perceptual load alters visual excitability. Journal of Experimental Psychology: Human Perception and performance 37(5), 1350–1360 (2011). 21728464 10.1037/a0024320

[b14] Macdonald J. S. P. & Lavie N. Load induced blindness. J. Exp. Psychol. Hum. Percept. Perform 34(5), 1078–1091. ISSN: 0096-1523 (2008). 18823196 10.1037/0096-1523.34.5.1078 PMC2672054

[b15] Cartwright-Finch U. & Lavie N. The role of perceptual load in Inattentional Blindness. Cognition 102(3), 321–340 (2006). 16480973 10.1016/j.cognition.2006.01.002

[b16] Lavie N. Distracted and confused? selective attention under load. Trends Cogn. Sci. 9, 75–82 (2005). 15668100 10.1016/j.tics.2004.12.004

[b17] De Haas B., Schwarzkopf D. S., Anderson E. J. & Rees G. Perceptual load affects spatial tuning of neuronal populations in human early visual cortex. Curr Biol. 24(2), R66–R67, doi: 10.1016/j.cub.2013.11.061 (2014). 24456976 PMC3928995

[b18] Palmer L., Schwarzkopf D. S., Moutsiana C. & Lavie N. Perceptual load degrades population orientation tuning in early visual cortex. Perception 44, 90–90 (2015).

[b19] Treisman A. & Gelade G. A feature-integration theory of attention. Cognitive Psychol 12(1), 97–136 (1980). 10.1016/0010-0285(80)90005-5 7351125

[b20] Wojciulik E. & Kanwisher N. The generality of parietal involvement in visual attention. Neuron 23, 747–764 (1999). 10482241 10.1016/s0896-6273(01)80033-7

[b21] Tomasi D., Ernst T., Caparelli E. C. & Chang L. Practice-induced changes of brain function during visual attention: a parametric fMRI study at 4 Tesla. Neuroimage 23, 1414–1421 (2004). 15589105 10.1016/j.neuroimage.2004.07.065

[b22] Wei P., Szameitat A. J., Müller H. J., Schubert T. & Zhou X. The neural correlates of perceptual load induced attentional selection: An fMRI study. Neuroscience 250, 372–380 (2013). 23876324 10.1016/j.neuroscience.2013.07.025

[b23] Culham J., Cavanagh P. & Kanwisher N. Attention response functions: characterizing brain areas using fMRI activation during parametric variations of attentional load. Neuron 32, 737–745 (2001). 11719212 10.1016/s0896-6273(01)00499-8

[b24] Jovicich J. *et al.* Brain areas specific for attentional load in a motion-tracking task. J. Cognitive Neurosci 13(8), 1048–1058 (2001). 10.1162/089892901753294347 11784443

[b25] Mitchell D. J. & Cusack R. Flexible, capacity-limited activity of posterior parietal cortex in perceptual as well as visual short-term memory tasks. Cereb Cortex (New York, N.Y., 1991) 18(8), 1788–1798, doi: 10.1093/cercor/bhm205 (2008). 18042643

[b26] Lavie N. & Fox E. The role of perceptual load in negative priming. J. Exp. Psychol. Hum. Percept. Perform 26, 1038–1052 (2000). 10884008 10.1037//0096-1523.26.3.1038

[b27] Lavie N. & Torralbo A. Dilution: a theoretical burden or just load? A reply to Tsal and Benoni (2010). J. Exp. Psychol. Hum. Percept. Perform 36(6), 1657–1664 (2010). 21133554 10.1037/a0020733 PMC3002221

[b28] Simons D. J. & Chabris C. F. Gorillas in our midst: Sustained inattentional blindness for dynamic events. Perception 28, 1059–1074 (1999). 10694957 10.1068/p281059

[b29] Somers D. C., Dale A. M., Seiffert A. E. & Tootell R. B. H. Functional MRI reveals spatially specific attentional modulation in human primary visual cortex. Proc. Natl. Acad. Sci. USA 96, 1663–1668 (1999). 9990081 10.1073/pnas.96.4.1663 PMC15552

[b30] Gazzaley A., Cooney J. W., McEvoy K., Knight R. & D’Esposito M. Top-down enhancement and suppression of the magnitude and speed of neural activity J. Cognitive Neurosci 17(3), 507–517 (2005). 10.1162/0898929053279522 15814009

[b31] Torralbo A. & Beck D. M. Perceptual load-induced selection as a result of local competitive interactions in visual cortex. Psychol Sci. 19, 1045–1050 (2008). 19000216 10.1111/j.1467-9280.2008.02197.x PMC2585548

[b32] Jeong S. K. & Xu Y. Neural representation of targets and distractors during visual object individuation and identification. J. Cognitive Neurosci 25, 117–126 (2013). 10.1162/jocn_a_00298 PMC3736830 23198893

[b33] Lavie N., Lin Z., Zokaei N. & Thoma V. The role of perceptual load in object recognition. J. Exp. Psychol. Hum. Percept. Perform 21(1), 42–57 (2009). 10.1037/a0016454 PMC2759815 19803641

[b34] Wei P., Kang G. & Zhou X. Attentional selection within and across hemispheres: Implications for the perceptual load theory. Experimental Brain Research 225(1), 37–45 (2013). 23187885 10.1007/s00221-012-3346-7

[b35] Alvarez G. A. & Cavanagh P. Independent resources for attentional tracking in the left and right visual hemifields. Psychol Sci. 16(8), 637–643 (2005). 16102067 10.1111/j.1467-9280.2005.01587.x

[b36] R Core Team R: A language and environment for statistical computing. R Foundation for Statistical Computing, Vienna, Austria. URL https://www.R-project.org/(2015).

[b37] Bates D., Maechler M., Bolker B. & Walker S. Fitting Linear Mixed-Effects Models Using lme4. Journal of Statistical Software 67(1), 1–48, doi: 10.18637/jss.v067.i01 (2015).

[b38] Kutnetsova A., Brockhoff P. B. & Chritensen R. H. B. LmerTest: Tests for random and fixed effects for linear mixed effect models (lmer objects of lme4 package). R package version (2013).

[b39] Sereno M. I. *et al.* Borders of multiple visual areas in human revealed by functional magnetic resonance imaging. Science 268, 889–893 (1995). 7754376 10.1126/science.7754376

