# Supplementary material for: Attention induced neural response trade-off in retinotopic cortex under load
Source: Sci Rep. 2016 Sep 14;6:33041. doi: 10.1038/srep33041 (PMC5021995; doi:10.1038/srep33041)
Supplement: Supplementary Information [file srep33041-s1.pdf]

Supplementary Information

Manuscript title:

Attention induced neural response trade-off in retinotopic cortex under load

Authors:

Ana Torralbo[1], Todd A. Kelley[1], Geraint Rees[1,2] and Nilli Lavie[1]

Affliations:

Institute of Cognitive Neuroscience, University College London, London, UK

Wellcome Trust Centre for Neuroimaging, University College London, London, WC1N 3BG,

Localizer and retinotopic procedures

Following the scanning experiment, participants underwent two runs of functional localizers blocks where four flickering black and white gratings and one flickering checkerboard were displayed in each block. Stimulated locations always changed in consecutive blocks (i.e. gratings appeared in the odd search locations and the checkerboard appeared in the right hemifield in one block while in the next block the gratings appeared in the even target locations and the checkerboard appeared in the left hemifield). Blank blocks lasted 16 s and localizer blocks lasted 22.8 s. 5 initial dummy volumes not used in the analysis were followed by a blank block, and four localizer blocks where two stimulation displays alternated. After a blank block, this sequence repeated with the remaining two stimulation displays, followed by a final blank. Participants received two runs with this structure where the displays seen in the second half in the first scan appeared in the first half in the second scan. Each localizer run lasted 250 seconds. Participants were instructed to maintain the eyes on the fixation cross and press a button every time they noticed a contrast change in the cross, which happened randomly once per block.

The retinotopy scans consisted of flickering checkerboard wedges presented alternatively at the horizontal and vertical meridians of the display for 22.8 seconds in each position. A 14 seconds blank block followed each stimulation block. Participants underwent a total of 5 blocks on each meridian condition per scan with a total of two scan sessions. They were instructed to keep the eyes at the fixation point throughout the whole scan. Each retinotopy scan lasted 376 s.

Regions of interest analysis procedure

Attended locations regions of interest (ROIs) were created by identifying the voxels that responded more selectively to each search location. We performed a differential localiser

where we contrasted blocks of odd search positions with blocks of even search positions (e.g. blocks of displays 1 and 4 with blocks of displays 2 and 3; see Figure S2). Four search locations are mapped in the left visual cortex and four are mapped in the right visual cortex. For each hemifield two search locations in the upper visual field are mapped in the ventral cortex and two target locations in the lower visual field are mapped in the dorsal cortex. Unattended locations ROIs where created by identifying voxels representing the left or right checkerboard such that we contrasted left checkerboard blocks with right checkerboard blocks (e.g. blocks of displays 1 and 3 with blocks of displays 2 and 4; see Figure S2). The localizers maps (thresholded at $p = 0.05$ uncorrected) were projected onto flattened representations of each participant's occipital cortex. Peak activation was identified in localizer maps within the V1v/d, V2v/d VP/V3 boundaries defined with our retinotopic mapping procedure. ROIs for every search location localizer (1 to 8) and every checkerboard localizer (left or right) were obtained by creating a 2D label representing contiguous activity, and were then imported into a 3D SPM8 ROI. In order to obtain the most selective voxels from the highest number of locations possible a functional voxel had to be filled at least 50% by the label to be included in the 3D SPM8 ROI (61% of the ROIs). If that threshold did not return any functional voxel for a label this threshold was lowered to 30% (applied to 33% ROIs) or 10% (5% of the ROIs) if the previous thresholds did not allow to obtain functional voxels from at least 60% of the ROIs from every participant map and visual area). There was no overlap between any of the ROIs.

Connectivity analysis

Psychophysiological interaction analyses were conducted where the seed regions were each participant's target V2-V3 ROI, each V2-V3 distractor ROI (composite images containing the left and right dorsal and ventral sections representing the checkerboards), and each of the fronto-parietal clusters obtained from the group level full brain analysis. The PPI function

from SPM was used to extract the first eigenvariate of the BOLD time course for each ROI and from each participant which was then convolved with a function representing the load effect (set size 5 > set size 2). Statistical Parametric Maps were obtained for each participant representing the level of effective coupling within each voxel. In a second stage, statistical maps were generated at group level using a t-contrast on the contrast images obtained from each participant, and were used to identify regions of functional connectivity with the seed regions. SPM maps were generated using a voxel-level threshold of $p < 0.005$ (uncorrected) and a family wise error correction at cluster level of $p< 0.002$ (corrected for multiple comparisons).

Behavioural task practice procedure

Prior to fMRI scanning participants received a practice session in a behavioural testing room to familiarize themselves with the task and the stimuli. Participants were instructed to perform the task as fast and as accurate as they can, while maintaining their eye fixation in the display centre (indicated with a fixation cross). A feedback in the form of a tone was given for incorrect responses or failures to make a response. Participants performed four blocks of 10 trials each (one per each load condition and an additional block with all load conditions intermixed) followed by one or two blocks with the same number of trials as the scanning runs. No feedback was given in the scanning experiment.

Retinotopic responses to target and distractor ROI: A re-analysis excluding trials with incorrect search responses

The data was reanalysed on the sub-set of trials after excluding all trials that resulted in an incorrect search task response (14% of all trials excluded). The results replicated the pattern of results found on all trials: The main effect of attention was significant in V1-V3 as before ($p = 0.02$ for V1 and $p = 0.003$ for V2, and $p = 0.008$ for V3) and importantly the

significant interactions of attention and load in V2 and V3 were also replicated (V2: $F(2,32) = 4.95$, $MSE = 0.298$, $p = 0.013$ V3: $F(2,32) = 8.94$, $MSE = 0.576$, $p < 0.001$), while the trend in V1 remained similar $F(2,32) = 2.30$, $MSE = 0.173$, $p = 0.116$. As in the full data the interaction also reflected increased target response and reduced distractor signal with high load.

Figure S1: Histograms of horizontal (top) and vertical (bottom) average eye position during the stimulus presentation interval for every set size across the experiment. Horizontal and vertical pupil position was determined for the 200 ms following the onset of the stimuli presentation in every trial, and converted to degrees of visual angle that deviated from fixation.

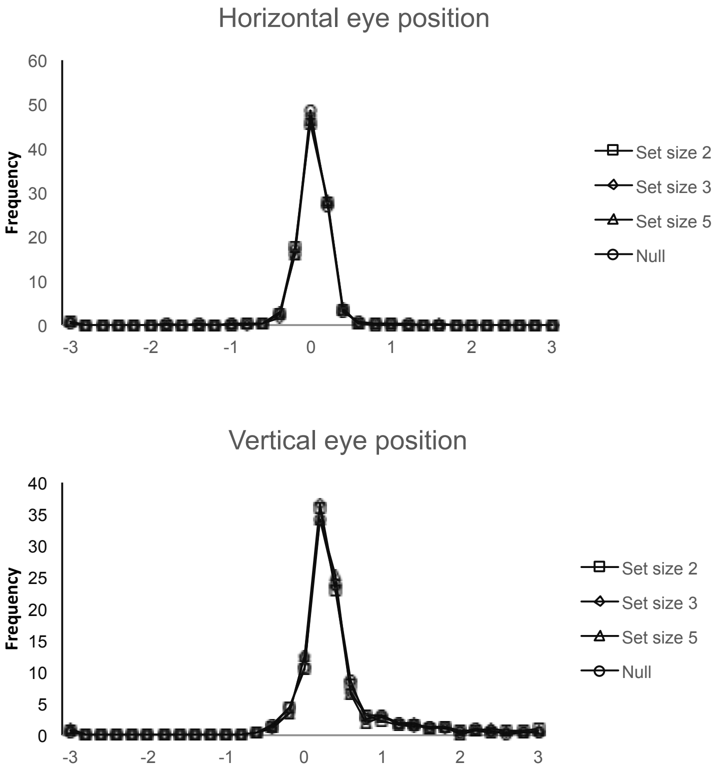

Figure S2. Localizer scans displays and activity maps overlaid in inflated surfaces of the visual cortex. Panel a. Example of stimuli used in the localizer scans. Flickering high contrast stimuli were placed in the task relevant and irrelevant stimuli locations. Panel b. Retinotopic mapping and functional localizer data are overlaid on an inflated surface of one participant's anatomical scan. 1: data from meridian mapping where warm colours represent BOLD response to horizontal meridian and cool colours represent BOLD response to vertical meridian. 2: data from localizer mapping to identify voxels that respond differentially to every search location. Figure depicts activation map overlaid on the inflated surface of the right hemisphere. Colours depict differential activity for search locations placed in the contralateral hemifield. 3: data from localizer mapping to identify voxels in right hemisphere that respond to the checkerboard placed in the contralateral visual field.

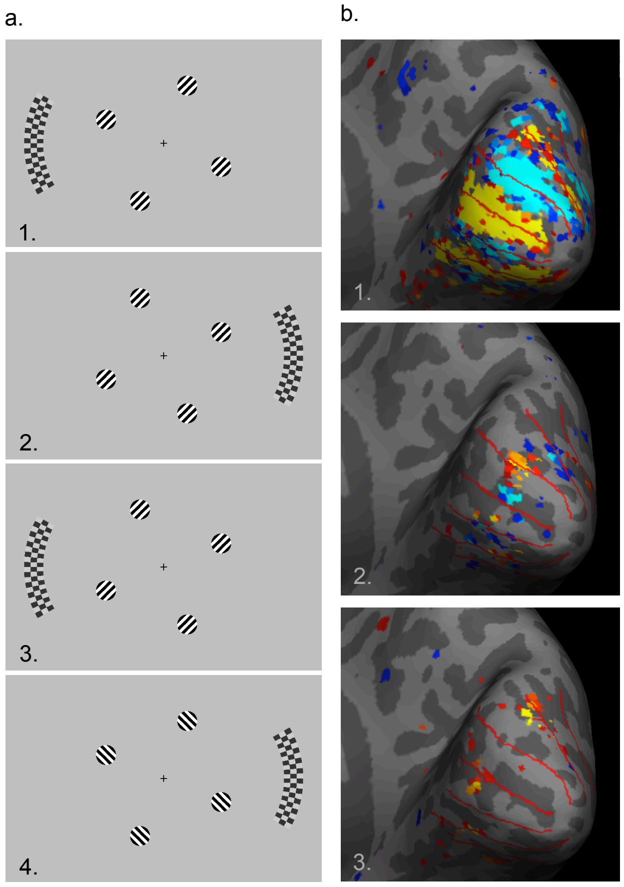

Table S1. Regions in visual cortex representing the target locations in the upper hemifield for both hemispheres and total of ROIs identified in each case out of the total number of participants in the study.

		V1v	V2v	VP
Hemisphere				
Left				
	location 1	9/17	9/17	7/17
	location 2	17/17	13/17	8/17
Right				
	location 7	11/17	12/17	6/17
	location 8	12/17	13/17	6/17

Table S2. Regions in visual cortex representing the target locations in the lower hemifield for both hemispheres and total of ROIs identified in each case out of the total number of participants in the study.

		V1d	V2d	V3
Hemisphere				
Left				
	location 3	14/17	14/17	8/17
	location 4	16/17	13/17	12/17
Right				
	location 5	16/17	14/17	10/17
	location 6	10/17	13/17	12/17

Table S3. Regions of visual cortex representing the distractor checkerboard locations in the right and left hemispheres and total of ROIs identified in each case out of the total number of participants in the study.

	V1v	V1d	V2v	V2d	V3	VP
Hemisphere						
Left	15/17	7/17	14/17	12/17	12/17	13/17
Right	17/17	14/17	13/17	12/17	12/17	11/17
